# The US Anti-Prostitution Pledge: Authors' Reply

**DOI:** 10.1371/journal.pmed.0040318

**Published:** 2007-10-30

**Authors:** Chris Beyrer, Nicole Masenior

We would like to thank Jay Silverman and Michele Decker for their thoughtful contribution to the public health discussion regarding commercial sex work and the grave issues of child prostitution and sex trafficking [1]. As the correspondents rightly assert, trafficking in persons is a criminal and human rights offense and should be vigorously opposed and its victims provided services. This is not a domain of any real contention in the public health or human rights communities. Nevertheless, the conflation of all forms of sex work with human trafficking, which they contend is an outcome of the essential inseparability of these two phenomena, does remain contentious. And, as with our paper, their work does not, arguably, resolve this contention. For the population of consenting adults who sell sex of their own volition, in settings as divergent from the India–Nepal context where Silverman et al. have worked as Washington D. C. or Amsterdam, sex workers and their advocates claim a domain of prevention and engagement that also uses human rights language, albeit the language of workers' rights and empowerment, to argue for services. And there is ample evidence to suggest that empowerment and an end to police harassment can improve health outcomes, including HIV.

It may be that the most important issues in sex work and sex trafficking are contextual. In Silverman and Decker's recent work, funded by the Office to Monitor and Combat Trafficking in Persons of the Bush Administration's Department of State, they reviewed medical documentation and case record materials for 287 sex-trafficked and repatriated Nepalese girls and women who received services at Maiti Nepal between January 1997 and December 2005. Their findings revealed that 109 (38.0%) women and girls were HIV positive [2]. In our own work, perhaps 60% of ethnic Shan women living in Burma who were trafficked to Thailand may be HIV infected [3]. In both these Asian communities, the contexts of poverty (Nepal and Burma relative to India and Thailand, respectively) and the very low status both of women and girls and illegal aliens more generally, makes it almost impossible for any sex work not to be profoundly exploitative.

In contrast, our work with AIDS service organizations in Moscow, Russia has taken place in a markedly different context [4]. The majority of women we encountered were adults when entering sex work, and over 80% indicated willingly entering into sex work and even coming to Moscow seeking such work. High rates of unemployment among women in the former Soviet Union, due to economic depression and gender discrimination, have brought these women to the Moscow sex trade seeking income. These economic factors are compounded by the fact that the majority of women in our sample were financially assisting or fully supporting family members. The fact that people besides themselves were dependent on the income of many of the sex workers must be taken into consideration in programs aimed at cessation of sex work. But none of the women interviewed were Moscow residents before entering the Moscow sex industry. Lack of legal status is perhaps the largest barrier preventing sex workers from receiving many important services and social benefits such as free and anonymous medical treatment, a steady job (outside of sex work), protection from the police, lodging, and psychological assistance. This is not a function of the legality of prostitution (sex work is not illegal in the Russian Federation), but of not having legal residency.

This example illustrates the highly contextual nature of sex work, and indeed, of trafficking, and perhaps argues against a “one size fits all” approach to these difficult problems.
